# Dual dose-dependent effects of fingolimod in a mouse model of Alzheimer’s disease

**DOI:** 10.1038/s41598-019-47287-1

**Published:** 2019-07-29

**Authors:** Isabel Carreras, Nurgul Aytan, Ji-Kyung Choi, Christina M. Tognoni, Neil W. Kowall, Bruce G. Jenkins, Alpaslan Dedeoglu

**Affiliations:** 1 Department of Veterans Affairs, VA Boston Healthcare System, 150 S Huntington Av, Boston, MA 02130 USA; 2 Department of Neurology, Boston University School of Medicine, 72 E Concord St, Boston, MA 02118 USA; 3 Department of Radiology, Massachusetts General Hospital and Harvard Medical School, 73 High St, Boston, MA 02114 USA

**Keywords:** Alzheimer's disease, Alzheimer's disease

## Abstract

Lipid metabolism is abnormal in Alzheimer’s disease (AD) brain leading to ceramide and sphingosine accumulation and reduced levels of brain sphingosine-1-phosphate (S1P). We hypothesize that changes in S1P signaling are central to the inflammatory and immune-pathogenesis of AD and the therapeutic benefits of fingolimod, a structural analog of sphingosine that is FDA approved for the treatment of multiple sclerosis. We recently reported that the neuroprotective effects of fingolimod in 5xFAD transgenic AD mice treated from 1–3 months of age were greater at 1 mg/kg/day than at 5 mg/kg/day. Here we performed a dose-response study using fingolimod from 0.03 to 1 mg/kg/day in 5xFAD mice treated from 1–8 months of age. At 1 mg/kg/day, fingolimod decreased both peripheral blood lymphocyte counts and brain Aβ levels, but at the lowest dose tested (0.03 mg/kg/day), we detected improved memory, decreased activation of brain microglia and astrocytes, and restored hippocampal levels of GABA and glycerophosphocholine with no effect on circulating lymphocyte counts. These findings suggests that, unlike the case in multiple sclerosis, fingolimod may potentially have therapeutic benefits in AD at low doses that do not affect peripheral lymphocyte function.

## Introduction

There is good evidence that lipid metabolism is abnormal in the brains of Alzheimer’s disease (AD) patients. These abnormalities affect glycerophospholipids like phosphatidylcholine and phosphatidylethanolamine, as well as sphingolipids. Abnormal sphingolipid metabolism in AD brains leads to the accumulation of proapoptotic and proinflammatory ceramides and sphingosine, while the level of sphingosine-1-phosphate (S1P) that enhances cell proliferation and antagonizes apoptosis decreases^1,2^. S1P levels are primarily regulated by two lipid kinases, the sphingosine kinase SPHK1 and SPHK2, and by the S1P-degrading enzymes, S1P phosphatases (SGPPs) that convert S1P to sphingosine and the S1P lyase (SPL), which irreversibly degrades S1P. During the progression of AD, S1P levels decline in a region-specific manner that correlates with the accumulation of β-amyloid (Aβ)^1^ and neurofibrillary tangles (NFT)^2^, the two pathological hallmarks of AD. The activity of sphingosine kinases (SPHK1 and SPHK2) also decrease with higher histopathological disease scores^2^. On the other hand, the level SGPP1 was shown to be markedly up-regulated in AD brains using microarray technology^3^. The biological functions of S1P have been linked to its ability to activate a family of five G protein-coupled S1P receptors 1–5 (S1PR1-5)^4^ that are widely expressed in the peripheral and central nervous system (CNS). Four of the five S1PRs (S1PR1, S1PR2, S1PR3, and S1PR5) are found in both neurons and glial cells^5^.

Fingolimod, a synthetic structural analog of sphingosine, is phosphorylated by SPHKs *in vi*vo and, when phosphorylated, is able to activate all S1PRs except S1PR2. Fingolimod was the first oral drug approved for the treatment of relapsing remitting multiple sclerosis (RRMS). Fingolimod binding to S1PR1 on lymphocytes prevents lymphocyte egress from lymphoid tissues and in RRMS, treatment with fingolimod reduces the infiltration of autoaggressive lymphocytes into the CNS, where they would cause inflammation and tissue damage^6^. Whereas S1P binding results in internalization and recycling of the S1PR1, phosphorylated fingolimod causes prolonged internalization and degradation of the receptor on lymphocytes, thus acting as a functional antagonist^7^. Moreover, fingolimod easily crosses the blood-brain-barrier and directly affects S1PRs on neurons^8^ and glial cells^9–11^. The direct actions of fingolimod in the CNS have been implicated in decreased production of proinflammatory cytokines^10^, enhanced expression of neurotrophic factors (especially brain derived neurotrophic factor, BDNF)^9^, enhanced synaptic function^12^, and anti-apoptotic activity^8^. Fingolimod has also been shown to reduce both the production^13^ and the neurotoxicity^8,14–16^ of Aβ peptides and to promote the survival of microglia^10^ and neurons^8^. We recently reported that 5xFAD mice treated with 1 mg/kg/day fingolimod resulted in decreased activation of microglia and reactive astrocytes, decreased accumulation of Aβ, and increased hippocampal neurogenesis compared to untreated transgenic littermate mice^17^. Since a dose of 1 mg/kg/day was more effective than 5 mg/kg/day, here we treated 5xFAD mice with fingolimod at 1, 0.3, 0.1, and 0.3 mg/kg/day from 1 to 8 months of age. We detected a dose-dependent decreased in the levels of Aβ in the hippocampus that correlated with the decreased peripheral lymphocyte counts. At 0.03 mg/kg/day, we detected improved learning and memory, decreased activation of microglia and astrocytes, and restored markers of neuronal GABAergic and cholinergic function.

## Results

### Effect of fingolimod treatment on learning and memory

**Figure 1:**
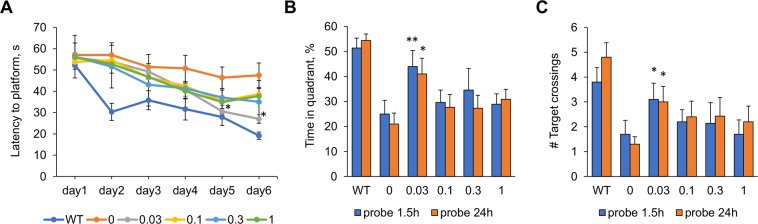
Effect of fingolimod treatments on learning and memory on the Morris water maze task. (**A**) Overall across training trials, 5xFAD mice treated with fingolimod (doses from 0.03 to 1 mg/kg/day) had a lower average latency to find the submerged platform compared to untreated 5xFAD mice. One-way ANOVA with Dunnett’s post hoc test revealed that WT mice showed more accurate learning than untreated 5xFADmice during days 2–6 of training. For 5xFAD mice treated with 0.03 mg/kg/day of fingolimod had a significantly lower escape latency on days 5 and 6 than untreated 5xFAD mice. During both memory probe trials performed 1.5 h and 24 h after the last training trial on day 6, 5xFAD mice treated with the 0.03 fingolimod dose spent significantly more time in the target quadrant where the platform used to be. (**B**) and similarly, 5xFAD mice from the 0.03 treatment condition crossed the area where the platform used to be significantly more times than untreated 5xFAD mice. (**C**) *p < 0.05, **p < 0.01 compared to samples from untreated 5xFAD mice (0 mg/kg/day).

We used the Morris water maze (MWM) behavioral task to assess the capacity of learning and memory of 5xFAD mice at 8 months of age and the effect of fingolimod treatment. As shown in the escape latency patterns in Fig. 1A, learning curves for fingolimod-treated 5xFAD mice lied halfway between the untreated 5xFAD mice and the non-transgenic mice (WT). Repeated ANOVA analyses for the escape latency revealed that during the 6 days of training there was a main effect of condition between subjects (*F*(5,52) = 7.92, *p* < 0.0001). Tukey post hoc analysis revealed that the learning curve of WT mice was significantly different than that of all the other subject groups. One-way ANOVA with Dunnett’s post hoc analysis using the untreated 5xFAD mice as a control showed that the escape latency of WT mice was significantly shorter than that of control mice in all six days of training, and that for mice treated with fingolimod at 0.03 mg/kg/day the escape latency was significantly shorter on day 5 and 6 than controls. No significant differences in escape latency were detected for any other dose. In the probe trials performed 1.5 h and 24 h after the last training trial, mice treated with fingolimod at 0.03 mg/kg/day significantly outperformed untreated 5xFAD mice on both probe trials: spending more time in the target quadrant where the platform used to be than untreated 5xFAD mice (Fig. 1B) (*p* < 0.01 for 1.5 hr probe; *p* < 0.05 for 24 hr probe) and had a greater number of passes through the platform location (Fig. 1C) (*p*’s < 0.05 for both probe tests). These results indicate that mice treated with 0.03 mg/kg/day of fingolimod from 1 to 8 months of age had more learning capacity and better short- and long-term memory than untreated 5xFAD mice.

### Effect of fingolimod treatment on the level of lymphocytes in blood

**Figure 2:**
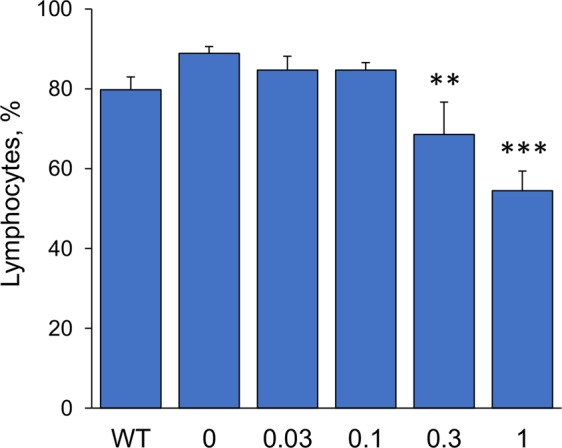
Dose-response effect of fingolimod on % of lymphocytes. The graph represents the percent number of lymphocytes amongst the total number of white blood cells. The oral treatment of 5xFAD mice with different doses of fingolimod from 1 to 8 months of age had a dose-dependent effect on lowering lymphocytes. The higher doses of 1 mg/kg/day and 0.3 mg/kg/day resulted in significantly decreased percentage of lymphocytes in blood. **p < 0.01. ***p < 0.0001 compared to samples from untreated 5xFAD mice (0 mg/kg/day).

To determine the dose-response effect of fingolimod on lymphocytes, blood samples collected at the time of death at 8 months of age were sent to Idexx Laboratories (Grafton, MA) for a complete blood count analysis. There was a significant effect of fingolimod treatment decreasing the percent number of lymphocyte in relation to the total number of white blood cells (*F* (4,42) = 12.90, *p* < 0.0001, one-way ANOVA) and a significant dose-dependent response detected by linear regression ANOVA (*F* (1,45) = 47.70, *p* < 0.0001) (Fig. 2). The Dunnett’s test, however, showed that only the two highest doses of fingolimod, 1 mg/kg/day and 0.3 mg/kg/day, resulted in significantly decreased percent number of blood lymphocytes compared to those in the untreated 5xFAD mouse group (*p* < 0.0001 for 1 mg/kg/day and *p* < 0.01 for 0.3 mg/kg/day).

### Effect of fingolimod treatment on Aβ accumulation

**Figure 3:**
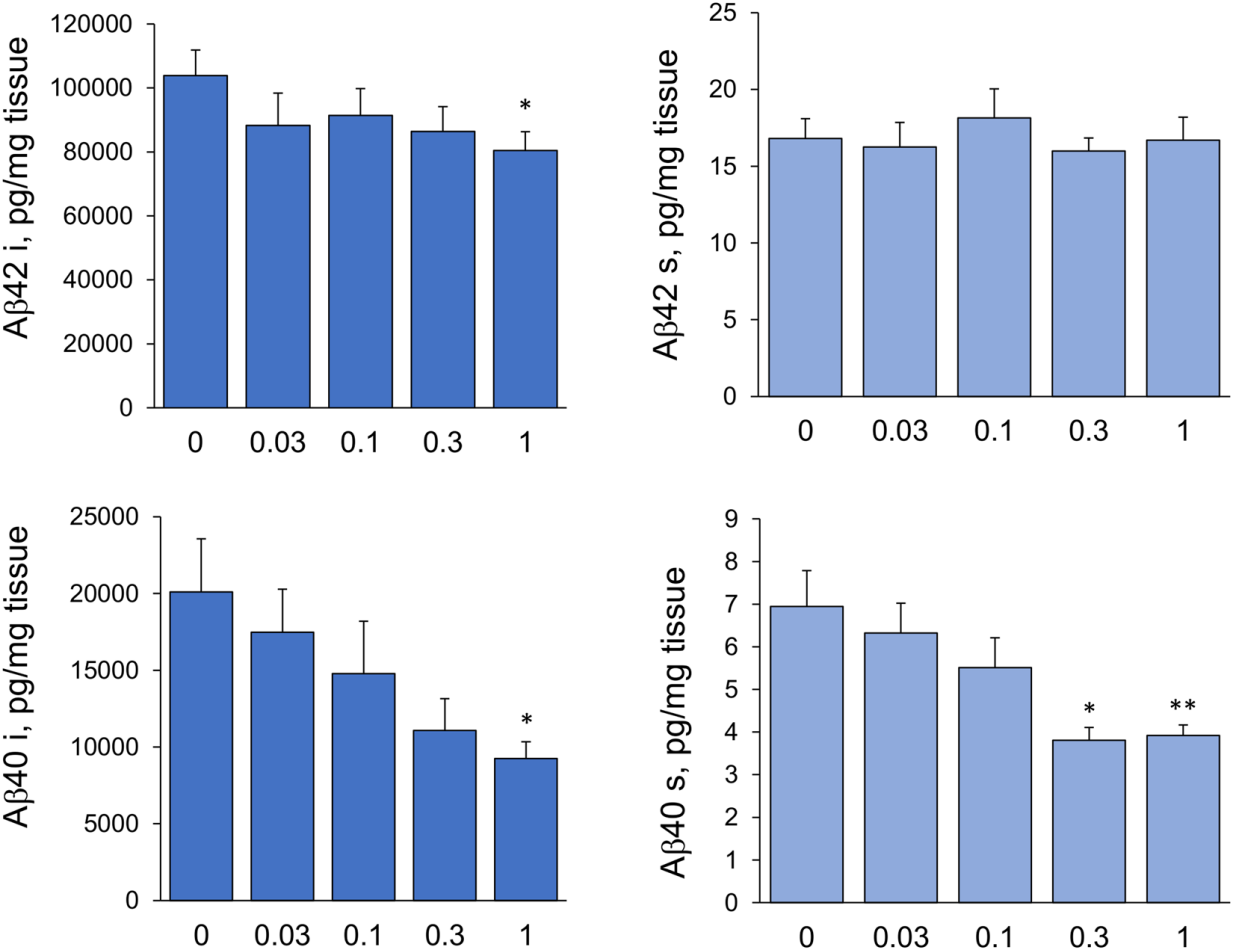
Effects of fingolimod treatment on the level of Aβ40 and Aβ42. Concentrations of soluble and insoluble Aβ40 and Aβ42 in the frontal cortex were quantified by ELISA. Oral fingolimod treatment of 5xFAD mice from 1–8 m of age had a dose-dependent effect on lowering the concentration of Aβ. The effect of fingolimod on Aβ was more prominent for Aβ40 than for Aβ42. (s = soluble; i = insoluble). *p < 0.05. **p < 0.01 compared to samples from untreated 5xFAD mice (0 mg/kg/day).

**Figure 4:**
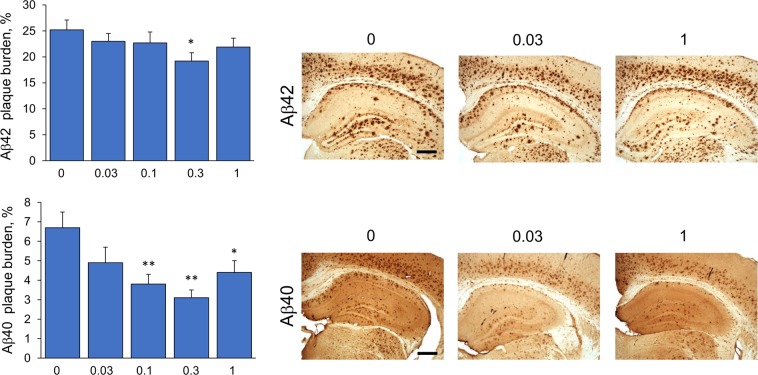
Immunohistochemical analysis of Aβ42 and Aβ40 in the subiculum/CA1 region of hippocampus. The subiculum/CA1 region was delineated using 40x magnification, and the percent plaque burden was calculated by densitometry analysis. Representative photomicrographs of tissue sections immunostained for Aβ42 and Aβ40 from untreated 5xFAD mice (0) and 5xFAD mice treated with fingolimod at the lowest (0.03 mg/kg/day) and highest (1 mg/kg/day) doses at 40x magnification are shown. Scale bar = 400 µm. *p < 0.05, **p < 0.01 compared to samples from untreated 5xFAD mice (0 mg/kg/day).

To evaluate the effect of fingolimod treatment on the level of Aβ in the brain, we measured the concentrations of Aβ42 and Aβ40 in the prefrontal cortex by ELISA (Fig. 3). We also quantified the percent area occupied by Aβ plaques in the subiculum/CA1 region of the hippocampus by densitometry analysis of Aβ42 and Aβ40 immunostained sections Fig. 4). Consistent with our previous studies^18^, 8-month-old 5xFAD mice had pronounced high levels of Aβ with higher levels of Aβ42 than Aβ40. We found a dose-dependent effect of fingolimod on the concentrations of insoluble and soluble Aβ40 (*p*’s < 0.01). The highest dose (1 mg/kg/day) resulted in a significant reduction of insoluble Aβ40 (*p* < 0.05), and both higher doses (1 and 0.3 mg/kg/day) significantly reduced of soluble Aβ40 (*p* < 0.01 for the 1 mg/kg/day and *p* < 0.05 for the 0.3 dose). We also detected reduced levels of insoluble Aβ42 in all fingolimod-treated mice but the effect only reached significance for the highest dose, 1 mg/kg/day (*p* < 0.05). There was a non-significant decrease in the Aβ40/42 ratio with higher doses of fingolimod (*p* = 0.08). Quantitative densitometric analyses of Aβ plaques in the subiculum/CA1 region showed that fingolimod treatment at doses of 1, 0.3 and 0.1 mg/kg/day resulted in a significant decrease on the percent area stained with antibodies to Aβ40; however with Aβ42 antibodies, similar to the ELISA results, the amount of plaques deposition did not significantly change with fingolimod treatment except for a decrease detected at a dose of 0.3 mg/kg/day.

### Effect of fingolimod treatment on microglia and astrocytes

**Figure 5:**
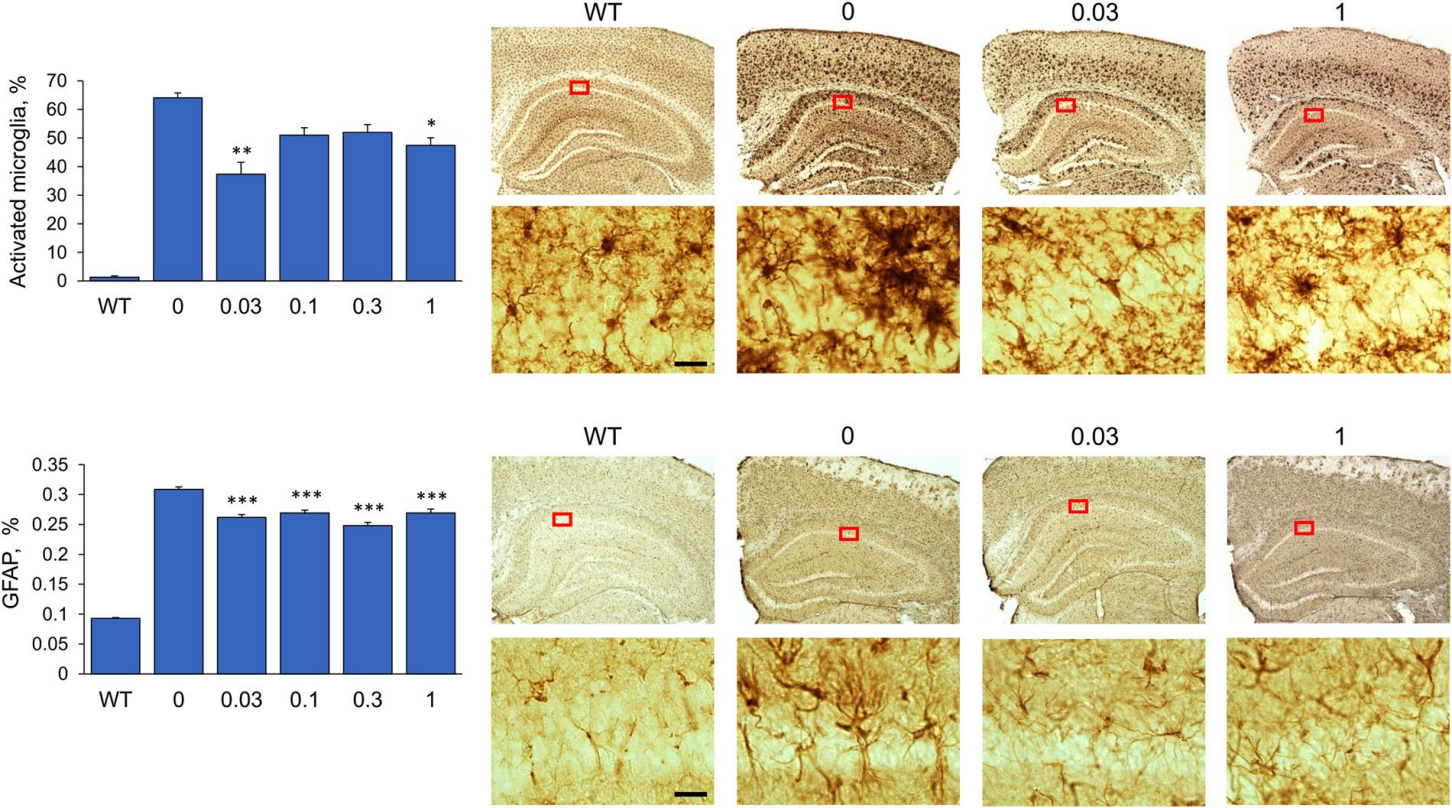
Immunohistochemical analysis of microglia activation and astrogliosis in the subiculum/CA1 region of hippocampus. Top panel shows the results from Iba1 immunostaining for microglia. Based on morphology, Iba1-positive cells were classified into activated microglia and resting microglia and counted using two different markers. Analysis of the percent (%) of activated out of total microglia showed decreased levels of microglia activation in fingolimod-treated 5xFAD mice. Significant reduction of microglia activation was detected for the doses of 0.03 and 1 mg/kg/day. The bottom panel shows the result of the densitometric analysis of brain sections immunostained for GFAP, a marker of reactive astroglia. All doses of fingolimod tested resulted in a similar highly significant reduction in the % of GFAP immunostaining. Representative photomicrographs of tissue sections immunostained for Iba1 and GFAP from WT, untreated 5xFAD mice (0), and 5xFAD mice treated with fingolimod at the lowest (0.03 mg/kg/day) and highest (1 mg/kg/day) doses are shown at 40x and 400x magnification. Rectangular insert indicates region magnified at 400x. Scale bar = 20 µm. *p < 0.05, **p < 0.01, ***p < 0.001 compared to samples from untreated 5xFAD mice (0 mg/kg/day).

We analyzed the effect of fingolimod treatments on the activation state of microglia and astrocytes in the hippocampal subiculum/CA1 region by immunostaining the brain tissue sections with antibodies to Iba1 and GFAP, respectively (Fig. 5). In AD and in mouse models of AD, microglia tend to concentrate in close proximity to amyloid plaques. Microglia associated with Aβ plaques display an activated phenotype characterized by enhanced Iba1-immunoreactivity, retracted processes, and perikaryal hypertrophy. This activated phenotype of microglia contrasts with that of microglia not associated with Aβ plaques, which majorly have lower level of Iba1 expression and display a resting morphology similar to the morphology of microglia under normal physiological conditions, with small compact soma bearing long, thin, and ramified processes. Based on these observed morphological differences (see the Material and Methods section for specific details), we counted the number of activated and resting microglia in the subiculum/CA1 region of Iba1 stained tissue sections and calculated de percent number of activated microglia. We detected a significant effect of fingolimod treatment on the percentage of activated microglia (*F*(4,40) = 12.00, *p* < 0.0001, one-way ANOVA). The percent of activated microglia was significantly diminished in mice treated with fingolimod at 1 mg/kg/day (*p* < 0.05) and at 0.03 mg/kg/day (*p* < 0.01) compared to untreated 5xFAD mice but not at the intermediate doses. Another pathological hallmark of AD is the presence of reactive astrocytes, characterized by hypertrophy and overexpression of the intermediate filament glial fibrillary acidic protein (GFAP). Densitometric analysis of the subiculum/CA1 region of GFAP immunostained brain sections indicated a significant effect of fingolimod treatment on decreasing the expression of GFAP (*F*(4,39) = 18.13, *p* < 0.0001, one-way ANOVA). All fingolimod doses reduced the percentage of GFAP immunostaining equally well (*p* < 0.0001).

### Effect of fingolimod treatment on the level of neurochemicals

**Figure 6:**
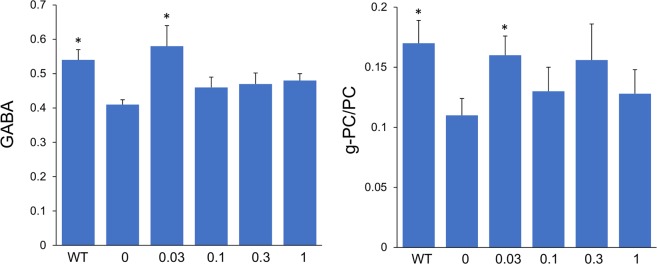
Effect of fingolimod treatment on brain neurochemicals measured by HRMAS. Fingolimod treatment at a dose of 0.03 mg/kg/day restored hippocampal levels of GABA (left) and g-PC/PC (right) to the levels of WT mice. Measures of GABA and g-PC/PC by HRMAS were normalized to the levels of creatine. *p < 0.05 compared to samples from untreated 5xFAD mice (0 mg/kg/day).

We have previously reported that 5xFAD mice at 8 months of age show numerous neurochemical alterations measured by proton magnetic resonance spectroscopy (^1^H-MRS) using the magic angle spinning spectroscopy (HRMAS) technique as compared to non-transgenic littermates using tissue punches from the cortex^18^. The HRMAS ^1^H-MRS changes include decreases in neuronal markers GABA, glutamate and NAA, and increases in glial markers glutamine and myo-inositol. Likewise, in the current study we analyzed tissue punches from the hippocampus and detected significant decreased levels of NAA and GABA and a significant increase in myo-inositol in untreated 5xFAD mice compared to WT littermates. Treatment of 5xFAD mice with 0.03 mg/kg/day of fingolimod – but not with any of other dose – led to a significant increase in GABA levels compared to untreated transgenic mice (*p* < 0.05) such that the GABA level was restored to that in WT mice (Fig. 6, left panel). In the HRMAS spectra we also detect three peaks for choline compounds - choline (C), phosphocholine (PC) and glycero-phosphocholine (g-PC) - although the latter two peaks overlap to some degree and thus we refer it as the combined g-PC/PC. In the hippocampus of untreated 5xFAD mice, we detected a significant decrease in the level of g-PC/PC compared to WT (Fig. 6, right panel). The decrease in g-PC/PC in the hippocampus contrasts with the increase of g-PC/PC detected previously in the cortex^18^. Similar to this finding in 5xFAD mice, a recent meta-analysis of over 600 patients with mild cognitive impairment (MCI) and 800 healthy controls, showed that the choline peak at about 3.2 ppm, which in human *in vivo* field strengths represents a mixture of the three choline compounds (C, g-PC, and PC) was increased in cortex and decreased in hippocampus of MCI patients who progressed to AD^19^. Similar to the effect of fingolimod on GABA, we detected significant higher levels of g-PC/PC in the hippocampus of 0.03 mg/kg/day fingolimod-treated mice compared to untreated 5xFAD mice (*p* < 0.05). The level of g-PC/PC in these treated 5xFAD mice was restored to the levels detected in WT (Fig. 6, right panel). No other metabolites showed significant change as a function of fingolimod treatment in the hippocampus.

### Data analysis (relief-f algorithm and linear discriminant analysis)

**Figure 7:**
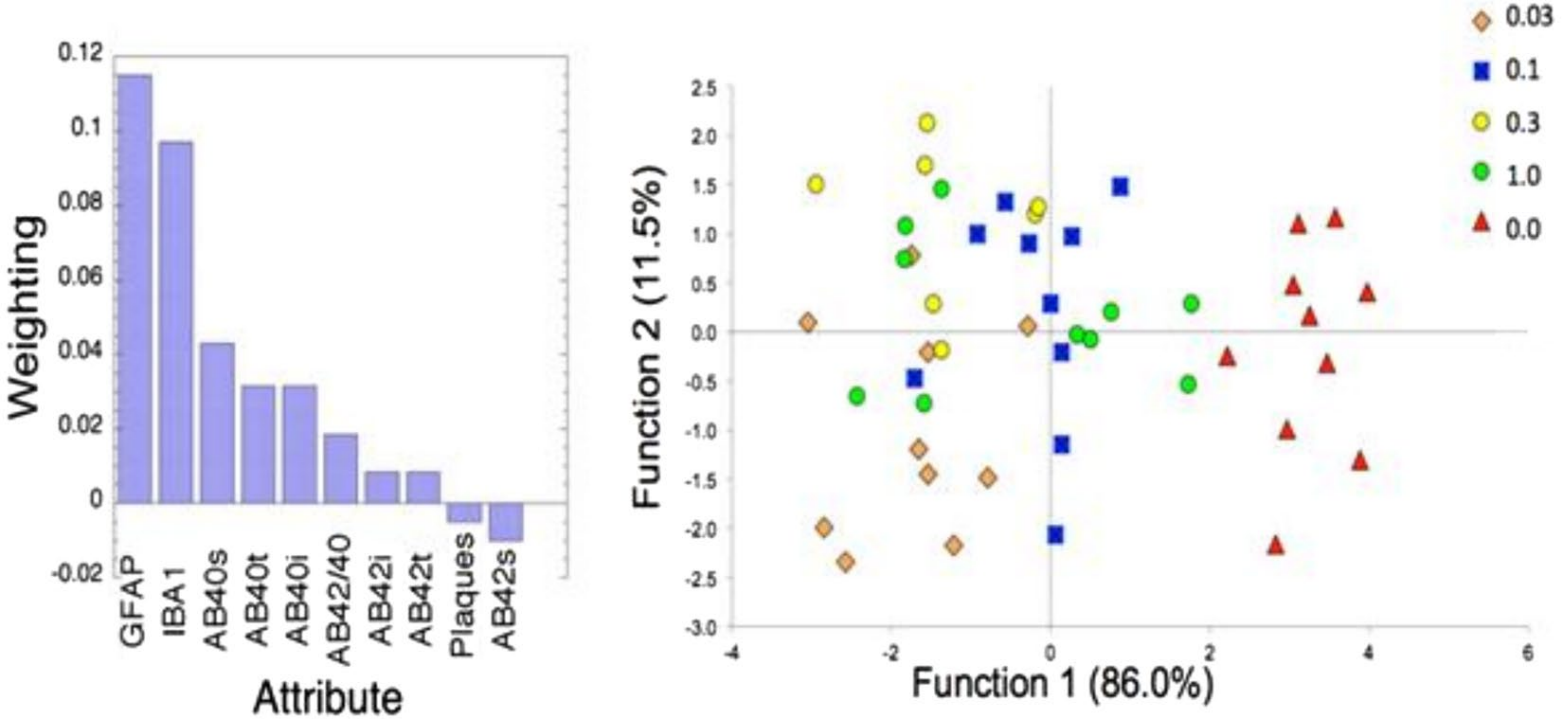
Machine learning effect size analyses of data. (Left) Rankings of parameters determined from a Relief-F algorithm (s = soluble; t = total; i = insoluble). GFAP had the highest weighting followed by Iba1. (Right) Linear discriminant analysis using the top two parameters (GFAP, Iba1) and Aβ40s and Aβ40/42 by fingolimod dose (Wilk’s lambda = 0.169; p < 0.0001 for function 1, 0.769, p < 0.006 for function 2). The largest average distance from control was for the 0.03 mg/kg/day dose of fingolimod.

Since brain samples from the same mice were analyzed by biochemical, immunohistochemical, and *in vitro* MRS assays, we used machine learning tools to reduce the dimensionality of the data set in order to identify those attributes that contributed the most to discriminate between the fingolimod-treated and untreated animals. In Fig. 7 (left panel) we present a plot showing the ranking of the various attributes using a Relief-F algorithm^20^. These data show that the results obtained from the quantification of activated microglia and reactive astrocytes measured in GFAP and Iba1 immunostained tissue outperform all the other metrics, providing evidence that the mechanism of action of fingolimod is essentially anti-inflammatory and target the brain’s innate immune cells. We then used the data from these two top markers (GFAP, Iba1) together with the data from soluble Aβ40 (Aβ40s) and the Aβ40/Aβ42 ratio to perform a linear discriminant analysis (Wilk’s lambda = 0.169; *p* < 0.0001 for function 1; 0.769, *p* < 0.006 for function 2). In Fig. 7 (right panel) we show the linear discriminant analysis for the different doses of fingolimod, which indicates that the best dose (shown by the cohort positioned at the farthest distance from the untreated transgenic mice) was 0.03 mg/kg/day. The distance of centroids from the untreated transgenic mice was 5 for the dose of 0.03 mg/kg/day and 3.5 for the dose of 1 mg/kg/day. This analysis suggests that the lowest dose of fingolimod was a better treatment overall than the highest dose. When we examined the behavioral metrics obtained from the Morris water maze task and the level of hippocampal neurochemicals measured by HRMAS, we also found that the dose of 0.03 mg/kg/day was the optimal dose. However, while the dose of 0.03 mg/kg/day fingolimod was the best in reducing the percent of activated microglia measured in Iba1 stained tissue, the reduction in GFAP immunostaining, a marker of astroglyosis, was equally strong for all of the fingolimod doses. A dose-response effect of fingolimod was detected for Aβ measurements with the highest dose of 1 mg/kg/day resulting in the most significantly decrease of Aβ levels in the prefrontal cortex. The best dose for the parameters that demonstrated significant treatment effects are presented in Table 1.

**Table 1:** Fingolimod dose at which a parameter demonstrated best significant treatment effect.

Parameters	Best dose (mg/kg/day)
GFAP	0.03, 0.1, 0.3, 1
Ibai1	0.03,
Learning and memory	0.03
GABA	0.03
g-PC/PC	0.03
Ab_40_ s,i,t	1.0
Lymphocytes	1.0

## Discussion

In our previous study on the effect of fingolimod on a transgenic mouse model of AD, 5xFAD mice were treated from 1 to 3 months of age, and our results indicated that a dose of 1 mg/kg/day was more effective that a dose of 5 mg/kg/day^17^. These results prompted us to study the effects of lower doses of fingolimod in 5xFAD mice treated with 0.03, 0.1, 0.3 and 1 mg/kg/day from 1 to 8 months of age. We found a dose-response relationship between fingolimod, the number of circulating blood lymphocytes, and brain Aβ levels. The highest dose, 1 mg/kg/day, had the largest effect on Aβ pathology, which may be mediated by reducing blood lymphocyte counts. The effect of fingolimod was more pronounced on Aβ40 than Aβ42. A previous study by Takesugi and colleagues^13^ reported a dose-dependent reduction of Aβ40 and Aβ42 by fingolimod in cultured neuronal cells. They suggested that the reduced production of Aβ was mediated by the inhibition of γ-secretase activity through a S1PR-independent mechanism. They also reported that while fingolimod reduced the level of Aβ40 in a transgenic APP mouse model it increased Aβ42 levels. It is intriguing that fingolimod appears to inhibit γ-secretase activity even though S1P has been shown to be a positive modulator of β-secretase^21^.

Although fingolimod reduced Aβ (and lymphocyte counts) at high doses, it was at the lowest dose tested (0.03 mg/kg/day) that fingolimod improved cognition and provided protection for the largest number of variables tested. Mice treated with 0.03 mg/kg/day of fingolimod had shorter latency to reach the platform than untreated transgenic mice during the last two days of training and demonstrated superior short and long-term memory in the probe trials. Since lowering lymphocyte levels in the elder population may have undesirable effects, our data suggest that fingolimod might be beneficial to Alzheimer’s disease patients at doses that do not alter peripheral blood lymphocyte counts. Given that one of the major adverse effects of γ-secretase inhibitors is reduced lymphocyte counts^22^, it may be that fingolimod could provide manifold protective benefits with reduced toxicity.

Our machine learning analysis shows that astrocytes and microglia effects are the greatest contributors to the efficacy of fingolimod, indicating that fingolimod’s mechanism of action in 5xFAD mice is essentially anti-inflammatory. Targeting the multifactorial effects of neuroinflammation could be advantageous in AD. As the brain’s resident immune cells, microglia is thought to regulate the degree of amyloid deposition by phagocytosis^23^ but chronic activation under increasing pathological conditions is associated with the production of neurotoxic inflammatory cytokines, reactive oxygen species^24^, and the phagocytosis of synapses^25^. Neuroinflammation also induces the efflux of Aβ and inflammatory mediators across the blood-brain barrier and the recruitment of leukocytes into the CNS that may contribute to the neurodegeneration, triggering a chronic inflammatory feedback loop^26,27^. Recent studies have provided overwhelming evidence of the involvement of microglia in the pathophysiology of AD^28^. Recently microglia that cluster around Aβ plaques have been found to have a novel neuroprotective effect by shielding neurons from Aβ neurotoxicity^29^. To this end it will be important to study specific phenotypic changes in microglia and whether fingolimod modulates the microglial response to amyloid plaques. Astrocytes may also contribute to cellular and functional degeneration in AD through disrupted glial–neuronal and glial–vascular signaling^30^. Consistent with our results showing a highly significant effect of fingolimod on astrocytes and microglia, numerous studies in primary cells and animal models neurological diseases indicate that microglia and astrocytes are central to the therapeutic effects of fingolimod^10,31–37^.

The suppressive effect of fingolimod on the activation of microglia was detected at the highest dose used (1 mg/kg/day) and even more significantly at the lowest dose (0.03 mg/kg/day) but not at intermediate doses. This suggests that the effects of fingolimod on microglia in 5xFAD mice may be mediated by two independent mechanisms. Decreased microglia activation by fingolimod at 1 mg/kg/day may be due to reduced Aβ levels, while effects at the 0.03 mg/kg/day dose may be due to activation of S1P receptors on microglia or by indirect effects mediated by astrocyte-derived factors. The protective effect of fingolimod appears to be greater on astrocytes than microglia because GFAP immunostaning was equally reduced in all doses tested. Although astrocytes and microglial cells both express S1P receptors and direct effects of fingolimod have been reported in both cell types, an indirect effect of fingolimod on microglia through the modulation of astrocyte-derived factors has been proposed by Hoffmann *et al*.^35^ based on a combination of *in vivo* and *in vitro* studies in a model of MS.

The effects of fingolimod on hippocampal neurochemicals was analyzed by HRMAS. This powerful technique can be directly applied to human studies for *in vivo* diagnosis and monitoring of disease progression and recovery. Using HRMAS, we found that the lowest dose of fingolimod tested (0.03 mg/kg/day) restored hippocampal GABA and g-PC/PC levels. The decrease in the g-PC/PC peak detected in 8-month-old 5xFAD mice was similar to what has been reported in the hippocampus of mild cognitive impairment patients who progressed to AD^19^. Abnormalities of choline and phosphatidylcholine metabolism in the brain and reduced basal forebrain cholinergic neurons (BFCN) function are found in AD patients^38–41^ and in animal models of AD^40,42–49^, and these instances of cholinergic system dysfunction are known to contribute to the memory deficits^40,41,50^. Importantly, BFCN also regulate neuroplasticity and facilitate functional recovery following brain injury^51^. Furthermore, brain derived neurotrophic factor (BDNF) is secreted by BFCN and is a neurotrophin involved in synaptic plasticity required for long-term memory^52^. Numerous studies have reported that fingolimod-mediated neuroprotection in associated with increased BDNF levels *in vitro* and *in vivo*^8,9,16,33,36,53–56^. As detected in the present study we have previously showed decreased level of GABA in 8 month old 5xFAD mice measured by ^1^H-MRS^17^ that was also reported by another independent laboratory^57^. A number of studies have shown that in human AD brain tissue (*in vivo* and post-mortem) there is decreased level of GABA^58–61^. The wide spread loss of GABA levels shown in these studies may simply be related to AD-associated neuronal loss. A recent study has shown that alteration of functional brain networks in AD are more related to GABAergic dysfunction than glutamatergic dysfunction in humans^62^. In an APPxPS1 mouse model of AD, Jo *et al*.^63^ showed an excess of GABA released from reactive astrocytes in the hippocampus that affected the spike probabilities of hippocampal neurons. Microdialysis measurements, as those reported in the cited publication, relates to extracellular GABA. The ^1^H-MRS measurements of GABA levels are primarily intraneuronal from both metabolic and neurotransmitter pools. The neurotransmitter pool is only about 20% of the total GABA concentrations. Therefore the increase of GABA reported by Jo *et al*. is not necessarily reflective of what is happening to tissue GABA levels as a whole. Nonetheless all the measurements in both humans and mice reflect GABAergic dysfunction associated with AD pathology and our ^1^H-MRS data suggest that fingolimod treatment is capable of correcting such dysfunction.

## Material and Methods

### Mice

Female 5xFAD transgenic mice and non-transgenic female littermates as controls were used in this study. 5xFAD mice coexpress the human APP and PS1 genes harboring a total of 5 FAD mutations [APP K670N/M671L (Swedish) + I716V (Florida) + V717I (London) and PS1 M146L + L286V] under the control of the murine Thy-1 promoter. The study comprised 4 groups 5xFAD mice treated with 4 different doses of fingolimod, untreated 5xFAD mice, and untreated non-transgenic (WT) mice (n = 10). No loss of mice occurred during the 7 months of treatment, and all 60 mice were included in the behavioral tests and histopathological analysis of blood and brain tissue. Mice were housed on a 12 h light:12 h dark schedule. All mice were given access to food and water ad libitum. All animal experiments were carried in accordance with the NIH Guide for the Care and Use of Laboratory Animals and were approved by research animal care committee at VA Boston Healthcare System.

### Drug protocol

Fingolimod was kindly provided by Novartis Pharma AG Basel, Switzerland. Fingolimod was dissolved in the drinking water at four different doses (1, 0.3, 0.1 and 0.03 mg/kg/day). Mice were treated for 7 months (from 1 to 8 months of age). Water was changed weekly. Food and water consumption were monitored weekly. We monitored mice for general well-being, and no side effects related to the treatments were observed.

### Morris water maze

MWM task was performed as previously described^64^. A white pool of 4 feet in diameter filled with water tinted with non-toxic white paint and maintained at 24 °C +/−2 was used. Mice were trained to find in the pool an invisible submerged platform using a variety of visual extra maze cues. Mice were trained in four trials per day of 60 s each during 6 consecutive days. Probe trials of 60 s without platform were run for all groups, 1.5 h and 24 h after the last raining trial. Data were recorded using an HVS 2020 automated tracking system (HVS Image, Hampton, UK).

### Tissue collection

Mice were euthanized by CO2 asphyxiation. Blood was collected onto heparin blood collection tubes and sent to Idexx laboratories for Cell Blood Count (CBC) analysis. The brain left hemisphere was post-fixed with the 4% paraformaldehyde solution for 24 h and cryoprotected in a graded series of 10% and 20% glycerol/2% DMSO solution for histological analysis. The right hemisphere was dissected at the frontal level (from bregma levels 3.2–1.3) and saved to prepared protein extracts for the analysis of Aβ by ELISA. The posterior hippocampus region (from bregma levels −2.7 to −3.7) was used to obtain a 1 mm diameter punch for analysis of neurochemicals by MRS.

### Enzyme-linked immunosorbent assay (ELISA) assay

Dissected frontal brain tissue was homogenized with 5 volumes (w/v) of Tris-buffered saline (TBS) and centrifuged at 100,000 g for 1 h at 4 °C. After saving the soluble supernatant fraction, the resulting pellet was resuspended with 8 volumes of cold 5 M guanidine HCl buffer and saved as the insoluble fraction. Specific human Aβ40 and Aβ42 ELISA kits (Invitrogen cat# KHB 3544 and KHB 3545) were used to analyze the soluble and insoluble tissue fractions according to the manufacturer’s specifications. Briefly, homogenized samples were added into the wells of a pre-treated 96-well plate, samples were then mixed with a cleavage-specific antibody to either Aβ40 or Aβ42. After an overnight incubation at 4 °C, plates were washed and incubated with the secondary antibody for 30 min at 25 °C. Washed wells were developed by the addition of a substrate. The substrate reaction was then stopped and color intensity was measured at 450 nm.

### Histology and immunohistochemistry

Cryoprotected hemibrains were serially cut at 50 μm on a freezing microtome. In brief, free-floating sections were incubated overnight in primary antibody followed by PBS (phosphate buffered saline) washes and incubation in peroxidase-conjugated secondary antibody followed by development using 3,3′ -diaminobenzidine tetrahydrochloride (DAB) as a chromagen. The antibodies used were ionized calcium binding adaptor molecule 1 (Iba1) (Wako Chemicals, cat# 019-19741; 1:5000) to stain microglia, glial fibrillary acidic protein (GFAP) (Chemicon, cat# MAB3402, 1:5000) to stain reactive astrocytes and Aβ 1–40 (Biolegend, cat# 805401, 1:400) and Aβ1–42 (Invitrogen, cat # 700254, 1:1000) to define Aβ40 and Aβ42 deposits. For each antibody we quantified, blindly to the treatment, the CA1/subiculum area of three serial sections/subject 0.5 mm apart from each other (from bregma −2.0 to −3.5). Quantification of GFAP and Aβ40 and Aβ42 immunostaining was performed by densitometry in 40 × images using a custom-made MATLAB program (Mathworks) that processes JPEG pictures of the region of interest and creates black and white images to provide percent burden of immunostainning. Each image was normalized for color and brightness using an unaffected region of the section. The percentage of thresholded pixels to total pixels in the region of interest was calculated for each image and presented as the percentage of affected tissue. Microglia activation was quantified at 400 × magnification using the Optical Fractionator probe in the Stereo Investigator software (MBF Bioscience, VT) with two different markers to classify and count resting microglia and activated microglia. Resting microglia was defined as a highly ramified Iba1-positive cell with a small soma of 10–12 μm exhibiting thin long primary processes with secondary and tertiary branches. As activated microglia we included hypertrophied and amoeboid Iba1-positive cell defined as having an enlarged, darkened soma and short, thick, and unbranched processes. The number of activated microglia expressed as a percentage of the total number of microglia was used as a measure for microglial activation.

## High Resolution Magic Angle Spinning Spectroscopy (HRMAS)

Proton magnetic resonance spectroscopy (^1^H-MRS) was collected as previously published^17^ using high resolution magic angle spinning (HRMAS) spectra on Bruker 14 T (Billerica, MA). We obtained tissue punches of freshly frozen hippocampus tissue. The dissected tissue sample was placed into a glass cylinder positioned in a 3 mm zirconium oxide MAS rotor (volume 50 μL). HRMAS measurements were performed using a sample spinning rate of 3.6 kHz selected to push the spinning side bands outside the frequency region of the metabolites. The experiments were performed at 4 °C to minimize tissue degradation. Data were acquired using a rotor synchronized, T_2_-filtered Carr–Purcell–Meiboom–Gill (CPMG) pulse sequence [90 − (τ − 180 − τ − Acq)_n_] with two different effective TEs (100 ms/10 ms). The longer TE serves to remove the lipid/macromolecular resonances and the short TE retains them. The interpulse delay, τ, is synchronized to the rotor frequency, and is 272μs. The n value for the relatively short T_2_ filter was 36 and for the long TE was 360. The short τ value removes all the T_2_^∗^ - like effects on the line shapes. The long T_2_ filter yields approximately 95% of the total spectral intensity of all metabolites of interest compared to the short TE. Other acquisition parameters were a 90° pulse of 5–10 μs, a spectral width of 8 kHz, 16 K complex points, 256 averages and a TR of 5 s. Samples were placed in the rotor with a small amount of D_2_O (Sigma-Aldrich, Milwaukee, WI) for locking and shimming. Data were analyzed using the Chenomx (Edmonton, Alberta, Canada) package fitting the entire metabolite spectrum for each neurochemical and reported as molar ratios to creatine. Classification of the data was performed using Weka^65^.

### Machine learning for discrimination between treatment groups

We utilized a Relieff algorithm to identify which parameters contributed the most to the separation between the groups. We also took the average values for the four most relevant parameters and determined which of the groups had the largest overall distance (D) from the average WT value by calculating D(i) = (V (i) − WT)/WT. D(i) is the distance for the parameter, and V(i) is the value of the parameter compared to the average WT value for each of the individual animals in the groups and averaging the numbers together. The data were further analyzed using well-established machine learning classification techniques to discriminate between groups including linear discriminant analysis (LDA), support vector machines (SVM) or multi-layer perceptrons (MP), all performed with hold-out analysis using the four highest ranked attributes to determine if there was a good classification between groups.

### Statistical analyses

Statistical analyses of the data were performed using one- or two-way ANOVAs with Tukey HSD post hoc or Dunnett’s post hoc as appropriate.
